# Workforce Wellbeing and Future Pandemic Preparedness in Emergency Departments: Lessons Learned While Caring for Persons Experiencing Homelessness During COVID-19

**DOI:** 10.3390/healthcare14142172

**Published:** 2026-07-18

**Authors:** Salima Bano Virani, Natalie Weiser, Melissa McGowan, Daniela Bellicoso

**Affiliations:** 1 Department of Emergency Medicine, St. Michael’s Hospital, Unity Health Toronto, Toronto, ON M5B 1W8, Canadamelissa.mcgowan@unityhealth.to (M.M.); 2 Interprofessional Practice Based Research, Unity Health Toronto, Toronto, ON M5C 3G7, Canada; 3 FIRST60: Prehospital, Trauma, & Resuscitation Science, Li Ka Shing Knowledge Institute, St. Michael’s Hospital, Unity Health Toronto, Toronto, ON M5B 1T8, Canada; 4 Li Ka Shing Institute, St. Michael’s Hospital, Unity Health Toronto, Toronto, ON M5B 1T8, Canada

**Keywords:** homelessness, workplace mental health, healthcare staff, emergency department, workforce planning, continuity of care

## Abstract

**Background/Objective**: Research has indicated that providing care during the COVID-19 pandemic negatively impacted healthcare providers’ wellbeing and burnout. Much of the literature has focused on emergency department (ED) physicians and nurses, including the distinct challenges they faced caring for persons experiencing homelessness (PEH), including addressing unmet basic needs, limited discharge options, and disruptions to community-based health and social services during the pandemic. Comparatively little research has examined the experiences of other multidisciplinary team members caring for PEH. This study explored the shared experiences of multidisciplinary ED staff caring for PEH during the COVID-19 pandemic to inform workforce planning and future pandemic preparedness. **Methods**: Using inductive qualitative analysis, we examined self-reported experience, mental health impacts and solutions for workforce planning and pandemic preparedness of multidisciplinary ED staff caring for PEH. Three virtual focus group discussions involving 12 multidisciplinary ED staff (registered nurses, clerical staff, security staff, community support workers, and a clinical assistant) were conducted during the first two years of the COVID-19 pandemic, a period characterized by evolving public health restrictions, uncertainty, and disruptions to health and social services. **Results**: Inductive qualitative analysis identified four key themes: (1) emotional experiences of care provision (including gratitude, compassion fatigue, and moral distress), (2) hospital- and community-led adaptations to support care delivery, (3) strategies for wellness and resilience building among staff, and (4) recommendations for future workforce planning and pandemic preparedness. **Conclusions**: Caring for PEH during the intense pandemic period intensified staff distress by exposing them to unmet social needs and system-level resource gaps, while also fostering interprofessional collaboration and resilience. Staff collaborated to facilitate organizational adaptations that enabled continuity-of-care. Future pandemic preparedness should include targeted mental health supports for staff, flexible institutional contingency planning, and formalized hospital–community partnerships to sustain care for PEH and other socially vulnerable populations.

## 1. Introduction

The COVID-19 pandemic established international consensus that recurrent pandemic infections are to be expected [1]. Pandemics carry various health, social, economic and political ramifications. From a healthcare organization perspective, they place significant strain on healthcare staff and systems. Frontline healthcare staff managing pandemics and providing patient care are at increased risk for mental distress and burnout [2]. Poor healthcare provider wellbeing reduces productivity and patient care due to absenteeism and presenteeism [3]. Following the COVID-19 pandemic, employee mental health and wellbeing has become a focal point for many organizations [4]. Prior to the COVID-19 pandemic, emergency department (ED) clinicians were already experiencing high levels of burnout, as demonstrated in pre-pandemic studies of ED physician and nursing populations [5]. Persons experiencing homelessness (PEH) commonly receive care through the ED, and caring for this population is associated with challenges for staff including burnout, mental distress and vicarious traumatization [6]. To foster workplace psychological safety for ED staff and support workforce planning and pandemic preparedness, the experiences of ED staff providing care to PEH under pandemic conditions warrants investigation.

PEH are at increased risk of COVID-19 infection due to various factors (e.g., shared housing, difficulties maintaining social distancing, high population turnover in shelters) [7,8]. Prior to the COVID-19 pandemic, PEH were found to utilize EDs at higher rates than the typical population; during the pandemic, they also accessed the ED at an increased rate [9,10].

Literature on the impact of previous infectious disease outbreaks on healthcare workers provides insight into the emotional, moral and psychological distress faced by frontline healthcare staff [2,11,12]. While pandemics have long occurred, the COVID-19 pandemic has built consensus on the recurrence of future pandemics, and the need to foster staff and organizational preparedness for future pandemics, in order to support resilience and continued functioning. Much of the research on ED staff mental health and wellbeing during the COVID-19 pandemic has focused primarily on burnout among physicians and nurses [13,14], with limited attention to the experiences of other ED staff who also play critical roles in caring for PEH, including clerical staff, security staff, clinical assistants, and community support workers. ED care for PEH is inherently multidisciplinary, requiring coordination among clinical and non-clinical staff, including physicians, nurses, allied health professionals, security personnel, and administrative staff. While individual professional roles differ, these groups operate within the same organizational environment and are collectively exposed to many of the structural and systemic challenges associated with providing care to this population. A multidisciplinary perspective therefore provides an opportunity to better understand shared organizational experiences and system-level factors that influence staff wellbeing and the delivery of care.

As a result, a gap remains in understanding how multidisciplinary ED teams, including both regulated and unregulated staff, experienced caring for PEH during the COVID-19 pandemic. This study examined the unique experience and reflections of a small group of frontline ED staff who interacted with PEH during the COVID-19 pandemic to understand how staff wellbeing was affected, and support workforce planning and pandemic preparation at the individual and organizational level. This study contributes to the literature by providing a view of the shared experiences of multidisciplinary ED staff caring for PEHs during the COVID-19 pandemic, with a particular focus on organizational and system-level factors influencing staff wellbeing and care delivery.

## 2. Methods

This study was conducted in the ED of a large inner-city academic health center providing tertiary and quaternary care, and housing a Level-1 Adult Trauma Center. This hospital serves a significant homeless population as it is located in the city’s downtown core and sees over 4000 ED visits annually specifically from PEHs [15].

An exploratory qualitative study design with focus group discussions (FGDs) was used to explore the experiences of ED staff who interacted with PEHs during the COVID-19 pandemic.

An email was sent to potential participants via the ED email listserv which included all ED staff (i.e., regulated patient care providers, clinical assistants, community support workers and hospital staff working on the unit and interacting with PEHs, such as clerical staff) working in this department. During the study period, the ED workforce comprised approximately 80 registered nurses (full-time, part-time, and casual), 30 physicians, 20 clerical staff, 20 clinical assistants, 20 security staff, and 2 community support workers. The email introduced the study and invited participants to contact the research coordinator for additional information and to be scheduled for a focus group. The research coordinator also introduced the study during education rounds. Purposive sampling was used to ensure the study sample represented a variety of occupational groups (i.e., regulated patient care providers, clinical assistants, community support works and hospital staff working on the unit and interacting with PEH). The study population also represented participants with varying years of experience (both in their profession and as employees of the organization), and those who work different shifts (i.e., day vs. night). Eligible participants were ED staff who were actively involved in providing direct care to, or supporting the care of PEH during the COVID-19 pandemic. Individuals not working in the ED, not involved in caring for PEH, or who did not provide informed consent were not eligible to participate.

Eligible participants took part in a focus group which was semi-structured in nature. Three focus groups were held between December 2021 and February 2022. A total of 12 participants were included in the study, comprising registered nurses (n = 3), security staff (n = 2), clerical staff (n = 4), community support workers (n = 2), and a clinical assistant (n = 1) Participants from multiple occupational groups were included in each focus group to facilitate discussion across diverse perspectives while maintaining participant confidentiality. The groups lasted an average of 45 min each.

Verbal consent was obtained from all participants at the time of the focus groups. All focus groups were conducted virtually through video-conferencing software (Zoom Healthcare Licensed Account©, Version 5.8) and recorded using the recording feature. The focus groups were conducted by trained interviewers with expertise in qualitative research (NW and MM). One team member conducted the interviews while a second took field notes. Using a semi-structured interview guide, interviewers actively encouraged contributions from all participants and used probing questions to elicit diverse perspectives and promote balanced participation. The interview guide included open-ended questions exploring participants’ experiences caring for PEH during the COVID-19 pandemic, perceived impacts on wellbeing and mental health, coping and resilience strategies, organizational adaptations, and recommendations for future workforce planning and pandemic preparedness. The focus group interview guide asked participants about their experiences and challenges caring for PEH during the COVID-19 pandemic, their need for and use of strategies during this time, and areas of improvement. Data collection and analysis occurred concurrently. Data saturation was determined through iterative team-based analysis, defined as the point at which no new codes, concepts, or thematic insights related to the shared experiences of multidisciplinary ED staff emerged across consecutive focus groups. This determination was made through ongoing comparison of emerging codes, review of field notes, and regular analytic team discussions. Focus groups were conducted until the research team determined that sufficient depth had been reached and no new information relevant to the study objectives was emerging. Consistent with qualitative content analysis, recruitment was intended to achieve sufficient depth to understand the phenomenon of interest across the multidisciplinary ED context, rather than to generate profession-specific findings.

Focus group audio recordings were transcribed verbatim. Data were analyzed using inductive qualitative content analysis informed by Elo and Kyngäs [16] within a constructivist/interpretivist framework. Transcripts were independently coded by two research team members (NW and SBV) using an inductive thematic coding approach to identify emerging patterns and themes [16]. Both researchers independently reviewed transcripts and met regularly to compare coding decisions and interpretations. Any differences in coding were discussed and resolved through consensus. An initial open coding process was used to generate preliminary codes, which were then iteratively refined into a structured coding framework and grouped into categories before final themes were developed. To enhance methodological rigor, analyst triangulation was used through independent coding by two researchers, and reflexive discussions were held throughout analysis to consider how researchers’ professional perspectives may have influenced interpretation. Codes were identified and organized into preliminary categories. The authorship team reviewed and refined initial categories and codes and used this coding structure to finalize the major themes which emerged from the data. Throughout analysis, the research team engaged in regular discussions to reflect on how their professional backgrounds and assumptions could influence interpretation of the data. Final themes were discussed with whole authorship team to ensure fidelity to the data.

### Ethical Considerations

This study received approval from the institutional Research Ethics Board (REB #21-250), and was conducted in accordance with the principles of the Declaration of Helsinki. All participants were provided with information about the study objectives, procedures, and voluntary nature of participation prior to enrollment. Verbal informed consent was obtained from all participants immediately prior to participation in the focus groups. Participants were informed that focus groups would be audio-recorded for transcription and analysis, and that all data would be de-identified to protect confidentiality.

## 3. Results

Twelve participants actively working in the hospital’s ED during the COVID-19 pandemic supporting or directly caring for PEH for the pandemic’s duration partook in the study (Table 1). Our inductive qualitative analysis identified four themes: the emotional experience of staff providing patient care in the ED, opportunities for hospital-led adaptations, strategies for building wellness and resilience, and pandemic preparedness suggestions (Table 2, Table 3, Table 4 and Table 5).

**The emotional experience of staff providing patient care in the ED**: The most pronounced feelings noted by staff providing or supporting care of PEH in the ED were gratitude and compassion fatigue (Table 2). To a lesser extent, feelings of fear of the unknown (e.g., lack of stability and predictability in the workplace, limited knowledge about the COVID virus), hopelessness and frustration with the then-current global healthcare situation, and grief for both the loss of life and hardships experienced by patients were also reported. Continuous changes to hospital routines and protocols to address pandemic needs (e.g., establish physical distancing, isolation) caused some confusion; however, participants also described appreciating leadership’s efforts to respond quickly to evolving circumstances and support teams caring for PEH. Participants described feeling recognized and appreciated when community members expressed gratitude for their work during the pandemic. For example, participants noted the morale boost they experienced when food and tokens of appreciation were donated to hospital staff from the community in recognition of their commitment to providing patient care. Several participants described these gestures as meaningful positive experiences during a period of uncertainty. Staff expressed appreciation for the comradery and bolstered sense of teamness that they felt amongst colleagues. Participants frequently described feeling closer to their colleagues and relying on one another for support during the shared experience of providing care throughout the pandemic. Compassion fatigue was commonly discussed in relation to witnessing the prolonged pain and suffering of patients, and was often intensified when participants described the unmet social and structural needs of PEH during the pandemic. For example, some staff noted the sense of devastation that came with being unable to recommend viable solutions to meet PEH’ basic needs (e.g., no shelter or place to safely lodge) after having listened to a patient’s story and experience. Participants often linked these feelings to repeatedly hearing patients’ stories, observing worsening circumstances, and feeling constrained by limited available. While staff reported experiencing compassion fatigue, they also described continuing to seek ways to support and care for PEH despite these challenges.

**Table 2:** Supporting quotes for Theme #1: Emotional Experience of Staff Providing Patient Care in the ED.

Emotional Experience of Staff Providing Patient Care in the ED
*[There is] always a fear of the unknown. You’re already anxious coming to work, what you can see. So aside from the volume, aside from thinking about what you’re going to do with your patients, but that time, it added more anxiety among us.* (FGD #1)
*This role came with this huge wave of grief of witnessing and seeing the pain and the people who were suffering. When there are no options and to walk a journey with someone and not have anywhere to recommend for them to go can be pretty devastating.* (FGD #3)
*The compassion fatigue part [really comes] into play when you already know the constraints of the system. But I think also constantly listening to some of our [PEH] that are newly homeless or have been clean for 15 years and now they’re back on substances…. I think, although we are there to work sometimes that kind of stuff cuts deep because you’re just doing your job. It’s been so stressful. You have that moment to take a pause and realize that this is actually a human being.* (FGD #3)
*I think it brought us [staff teams] a lot closer. You know, it was difficult, because a lot of our families didn’t really want to get together with us. And we didn’t want to get together with them, just so that we would protect them. And so it was nice to like, be in that environment where we had each other.* (FGD #1)
*At the very beginning, when we were getting the food brought to us, the snacks and the gifts from outside the hospital, I thought that was really good.* (FGD #2)
*The thing that I came up with was just trying to take home all the positive and just take one day at a time for me when the day is over. You know, I reflect back and, you know, take all the good things from the lessons I’ve learned. And I think about what I could do tomorrow.* (FGD # 3)

**Opportunities for Hospital-Led Adaptions:** The second theme respondents raised was regarding hospital-led adaptations in the pandemic context (Table 3). Participants reflected that the pandemic necessitated adaptations to ED spaces and processes to optimally utilize ED space to further support PEH. In particular, staff noted examples such as converting the waiting room and old trauma area to makeshift shelter space as well as an additional ED-specific COVID Assessment Center.

Pandemic restrictions limited access to community-resources (e.g., safe injection sites, primary healthcare and drop-in centers). Closure of these resources prevented PEH from using the intended resource (e.g., accessing care), and accessing secondary supports they once provided (e.g., washrooms), resulting in more people accessing the ED for basic needs. Participants described instances in which the hospital provided food, shelter, and other basic supports when community resources were unavailable. Participants highlighted that gaps in community resources were creatively filled by the hospital (e.g., providing food to PEH, shelter, care for basic needs). The hospital and representatives from the community worked together to establish resources to meet the needs of PEH (e.g., temporary housing by converting hotel rooms, identifying spaces and opportunities where care could be provided in the community and facilitating safe transfer between the hospital and temporary community lodging) thus highlighting the importance of hospital and community partnership and co-planning. While these adaptations enabled continuity of care during acute system disruption, participants also described them as time-limited and reactive measures, raising questions about their sustainability and the extent to which they addressed underlying structural gaps in community-based services.

**Table 3:** Supporting quotes for Theme #2: Hospital and community level adaptations in the pandemic context.

Hospital and Community Level Adaptations in the Pandemic Context
*I think that one of the biggest issues was space in the ED. Where do we put them [PEH] in order to follow social distance guidelines? They can’t walk around. The hospital needs to have some alternate space which may not be in the emergency department… We converted the waiting room into a makeshift shelter [in under 24 h]. The old trauma room was also converted to a COVID Assessment Center in the first wave.* (FGD #1)
*We are always really good at adapting. So we adapted a fair bit right away to make up for absences [e.g., shelters, comfort stations] we were noticing in the community in order to help our patients who were experiencing homelessness.* (FGD #2)
*There was a lot of recognition that the system as a whole had gaps, but when it was within the ED’s capacity, they found opportunities to support the patient.* (FGD #3)
*I think community partnerships are important because we’re serving the same community and often doing so in silos.* (FGD #1)

**Strategies for building wellness and resilience:** The third theme referenced supporting staff mental health and using wellness and resilience strategies (Table 4). Participants described coping strategies they employed during the pandemic and supports that bolstered their mental health. Many noted that community support, tokens and signs of gratitude offered during the earlier pandemic months were highly valued by ED staff with participants describing these gestures as helping them feel recognized and appreciated. While it was understood that these supports could not continue indefinitely, there was a sense of feeling forgotten by the public as recognition tapered over time, yet ED staff were still caring for and managing patients affected by the pandemic.

Participants frequently described colleagues as an important source of support during the pandemic. Participants described co-workers as a family with a shared lived experience of interacting with and providing care for PEH as only those working inside the hospital could fully understand and relate to. This shared experience was described as fostering camaraderie and providing opportunities to discuss workplace challenges with colleagues who understood their circumstances.

Respondents described self-care techniques including intentionally celebrating small wins or milestones at work, not taking work stresses home and completing hobbies. Participants described recognizing small successes, such as securing shelter for a patient, as helpful during challenging work shifts. Respondents noted that they reflected on the good things that happened during their work shifts, and thought about strategies for improvement to build resilience. Staff reported that they engaged in activities that brought them joy (e.g., hobbies, personal time) outside of work as additional self-care strategies. These accounts align with resilience frameworks that conceptualize peer support and informal coping strategies as key protective factors in high-stress healthcare environments, although they also highlight the reliance on informal rather than institutionalized supports.

**Table 4:** Supporting quotes for Theme #3: Supporting mental health with wellness and resilience strategies.

Supporting Mental Health with Wellness and Resilience Strategies
*The community was incredibly generous to the emergency department and the entire organization through the first wave and second wave. We were very fortunate to get pretty much almost every meal provided to us through various donations and restaurants.* (FGD #1)
*When that [food donations from community] kind of stopped, and everything kind of dragged on, you kind of felt like you’re forgotten.* (FGD #2)
*I think it definitely brought us [the staff] closer. I think that we kind of just used each other as our therapists. A lot of people weren’t even going home, we [the staff] were just sleeping at the hospital in the rooms or classrooms provided to us at the organization.* (FGD #1)
*I say it’s your fellow staff that you look forward to going to work and talking to certain people because they are the ones who can relate to what we’re going through. That is your confidant for getting through all the tough times.* (FGD #2)
*I try to celebrate small successes, it might be that we found or secured a shelter for someone. I try to relish in those rainbows with small things, those small wins. It gives you strength to get through.* (FGD #3)
*You know, I reflect back and […] take all the good things from the lessons I’ve learned. And I think about what I could do tomorrow* (FGD #3)
*When I’m at home, I try to do things that I like to do. I try to keep myself occupied, I cook and I try to make music.* (FGD #2)

**Pandemic preparedness suggestions:** The fourth theme was pandemic preparedness and workforce planning, with participants offering suggestions based on their experiences during the COVID-19 pandemic (Table 5).

Participants emphasized the importance of psychologically safe work environments where ED staff could openly discuss concerns related to wellbeing (e.g., compassion fatigue, anxiety) and access support. Participants also highlighted communication, peer support, and collaboration within and across hospital teams when discussing future pandemic preparedness. The creation of internal hospital contingency plans and strong external partnerships with community organizations which serve PEH were recommended to ensure continuity of care and resources during pandemics.

**Table 5:** Theme #4: Suggestions for future pandemic preparedness, and supporting quotes.

Suggestions for Workforce Planning and Future Pandemic Preparedness
*It was nice to be in that environment where we had each other to support or share our feelings on being scared or anxious and just literally became each other’s therapist.* (FGD #1)
*For future, it would be invaluable to have someone who has been educated and prepped on what needs to be done, what the backup plans are, such as what plan C, D and E look like. As the pandemic was such an unknown experience, everything was changing so rapidly* (FGD #1)
*And there’s always more that we could do. But no matter how much we do, I think at the end of the day, it’s extremely important to understand and realize that we as one organization cannot do everything alone. So making those partnerships, reaching out to the community and tapping those resources…* (FGD #3)
*Community partnerships are important because we’re serving the same community and often doing so in silos. So creating alliances and relationships with other organizations will enhance the service that people get and the institutional memory between both spaces. It broadens what we can offer people to do* (FGD #3)

## 4. Discussion

Our study of a small cohort of multidisciplinary healthcare providers in the ED identified several factors that can support the care of PEH during the context of a pandemic. These factors included strategies to support staff recognition, resilience and coping, along with institutional adaptations and resources, and community partnerships to support PEH. Workforce preparedness suggestions emerged in line with the belief cemented by the COVID-19 pandemic that epidemics and pandemics will continue to occur worldwide and contingency planning is required to support continuity of care [1]. Workforce and pandemic preparedness planning can impact staff wellbeing under pandemic conditions, and improve organizational and community protocols to facilitate delivery of care to PEH. Published research highlights the need for healthcare workforce planning to support patient care [17,18]. Our study contributes to the growing literature on workforce planning by highlighting the need to implement personal and organizational strategies that may benefit staff well-being while caring for PEH during public health emergencies. Advanced planning and strategizing is needed to ensure resilience at the individual level, the healthcare organization level and the community level. Although these findings emerged during the early phases of the COVID-19 pandemic, they highlight persistent system-level vulnerabilities including workforce strain, fragmented social supports, and the dependence of vulnerable populations on EDs that remain relevant to contemporary emergency care and future public health crises.

ED staff caring for PEH during the COVID-19 pandemic reported various emotional experiences. Feelings of confusion or fear of the unknown stemmed from limited knowledge about the then-new virus, as well as from continuous change, however a sense of appreciation was also linked with the feelings of confusion. Respondents noted they felt grateful for immediate support from leadership to guide solutions to support the care of PEHs, and for quick action from colleagues to collaboratively implement novel care plans. Under the novel and trying pandemic circumstances, collegial attitudes and willingness to work towards solutions counterbalanced less positive emotions, and allowed staff to experience uplifting feelings of appreciation and gratitude. Under pandemic and non-pandemic situations, supportive leadership and collegiality to work towards shared goals both have the potential to help staff achieve positive emotional experiences in the workplace.

Although many of the emotional responses described by participants, including uncertainty, fear, and grief, were also reported more broadly among healthcare workers during the COVID-19 pandemic, participants described these experiences within the context of caring for PEHs in the ED. The intent of this study was not to distinguish pandemic-related stressors and those associated with caring for PEH in the ED, but rather to understand how participants experienced these intersecting challenges. Our findings suggest that participants perceived pandemic-related pressures and the challenges associated with caring for PEH as closely interconnected aspects of their experiences of working in the ED, rather than as discrete or mutually exclusive phenomena.

Compassion fatigue (negative or stressful feelings stemming from providing care to patients who have experienced grave illness and or trauma [19]) was reported by respondents, highlighting the emotional toll of caring for PEH during the pandemic and demonstrating staff’s ongoing investment in their patients’ wellbeing even when they themselves were operating under difficult pandemic-related circumstances. Aligned with compassion fatigue, hopelessness and grief for the wellbeing and loss of life of PEH was noted. Compassion fatigue was noted across FGDs, indicating ED staff providing direct or supportive patient care (e.g., nurses versus clerical or security staff) are impacted by the emotional toll of interacting with patients experiencing marginalization and homelessness. While this research examined the impact of providing care for PEH in the ED during a pandemic, the phenomenon should also be examined under non-pandemic conditions, given that PEH often access care via the ED at a higher rate than persons not experiencing homelessness [20,21]. Future research should examine the overarching construct of burnout (and the positive compassion satisfaction component [19]) among ED and healthcare staff interacting with and caring for PEH.

Participants’ accounts also reflected elements of moral distress, particularly when they described knowing what patients experiencing homelessness needed but being unable to secure appropriate shelter, housing, or community supports due to pandemic-related service disruptions. Moral distress refers to the psychological distress that arises when individuals recognize the ethically appropriate action but are constrained from acting upon it because of institutional, structural, or resource limitations. In this study, participants frequently described feelings of frustration, helplessness, and devastation when attempting to support patients whose basic needs could not be met despite their efforts. These experiences suggest that moral distress may have been amplified by the intersection of pandemic-related system pressures and longstanding structural inequities affecting PEH. Similar findings have been reported among healthcare workers during COVID-19, where resource limitations and barriers to providing optimal care contributed to emotional exhaustion and psychological strain. Recognizing and addressing moral distress may therefore be an important component of future workforce wellbeing initiatives, particularly for staff working with structurally vulnerable populations.

Future pandemic planning to support workplace wellbeing among ED staff and hospital employees at large should consider supports respondents indicated aided their wellness and resilience. In accordance with research conducted under non-pandemic conditions supporting the benefit of healthcare staff recognition to boost morale, job satisfaction, and workplace mental health [22] providing recognition (i.e., helping staff feel seen) can be explored under pandemic conditions to identify effective solutions. The need for continued peer support and building safe spaces to discuss workplace psychosocial experience should be explored; this need has been highlighted by previous research (predominately examining nurses and physicians) prior to and during the COVID-19 pandemic that highlights the benefit of being able to safely turn to workplace peers who have shared the same emotionally tiring experience [23,24]. Aligned with respondents’ feedback to support teamness and communication strategies for future pandemic preparedness, these variables could be incorporated to build peer support.

Respondents highlighted self-care, which can take various forms and occur on a personal level or be supported at an organizational level through reflection and resilience building initiatives [25,26] improved their mental health and resilience. Nurturing resilience among ED staff and across the organization, prior to and during pandemics, is important as it has been shown to reduce burnout (including in high intensity healthcare settings) and its subsequent negative impacts on patient care and satisfaction [27,28,29,30].

Hospital-led adaptations highlighted a challenge that may be particularly relevant to caring for PEH during pandemics. When community-based supports are restricted, EDs may become responsible for addressing both acute healthcare needs and basic social needs such as shelter, food access, hygiene, and care coordination. Within the ED, workforce planning should ensure staff can adequately provide care and direct PEH to resources they rely upon for survival (e.g., establishing policies on how to rapidly mobilize ED, hospital, or specially designated space as a shelter and how care can be provided in this circumstance, identifying procedures for how to rapidly screen and isolate PEH when necessary to reduce virus transmission, developing contingency plans in partnership with the community to safely house PEH). Recommendations for improved hospital–community partnerships to support care for PEH aligns with preliminary research conducted early in the COVID-19 pandemic that demonstrated hospitals with greater community partnerships experienced reduced case-fatality rates, and the particular benefit of these relationships for providing care to vulnerable populations [31]. Partnerships should be explored between healthcare organizations and community resources to understand how services that might be restricted under pandemic conditions (e.g., safe injection sites, primary care), can be offered in alternate formats, or how PEH can be best supported. Such solutions could help ED staff feel equipped to adequately direct and support PEH who were experiencing diminished wellbeing. In addition, public outreach strategies for PEH could support pandemic protocol awareness and understanding (e.g., masking, social distancing, vaccination), and ensure their well-being within the community (e.g., encouraging vaccination).

Importantly, our findings indicate that some preparedness recommendations are broadly applicable to supporting healthcare staff during pandemics, whereas others are uniquely informed by caring for PEH in the ED. General workforce preparedness measures included fostering psychological safety, promoting peer support, recognizing staff contributions, and supporting resilience-building activities. In contrast, PEH-specific preparedness considerations focused on maintaining access to basic needs and community services during public health emergencies. To facilitate translation of the study findings into practice, Table 6 summarizes how the identified themes can inform workforce planning and pandemic preparedness actions.

Importantly, participants described challenges arising when shelters, drop-in centers, harm reduction services, primary care, and public facilities became less accessible, increasing reliance on the ED for both healthcare and social support. These findings suggest that pandemic preparedness planning for PEH should extend beyond hospital operations alone and include coordinated contingency planning with community partners to ensure continuity of housing, harm reduction, and social supports. Findings in this study also underscore that pandemic-related disruptions to community-based services disproportionately affected PEH, reinforcing the importance of integrated hospital–community responses in future public health emergencies.

### Limitations

The main limitation of our study was the small sample size (n = 12). While a wider group of participants was approached to participate in the study, the smaller sample size is suggestive of the burden many ED staff experienced that limited their ability to partake. Nonetheless, the study participants helped to elucidate practical strategies relevant to future pandemic planning and future studies could include a broader representation of healthcare providers to identify any possible missed opportunities. Our study took a multidisciplinary approach and incorporated a variety of regulated and unregulated ED staff in different roles all caring for PEH. Our results indicate that mental health impacts of caring for PEH are shared across healthcare staff, and that the same required supports and potential solutions (personal, organizational, and community-level) exist across these staff. Social-distancing restrictions within our organization at the time of data collection required FGDs to occur via Zoom©, which was challenging for some participants who were unfamiliar with its use. This may have impacted their ability to participate in a more fulsome capacity, but overall, many of the same sentiments and reflections were shared across respondents in each FGD.

As with all self-reported qualitative research, there is a possibility of social desirability bias. Participants may have been reluctant to criticize organizational responses to the pandemic or may have emphasized positive coping strategies and adaptations due to their affiliation with the organization. Although participants were assured of confidentiality and focus groups were facilitated by experienced qualitative researchers, these factors may have influenced how experiences were described. In addition, this study was conducted within a single urban academic ED with a longstanding commitment to serving PEH. Organizational culture, available resources, and local community partnerships may differ from those in other settings, which may limit transferability of findings. Finally, several members of the research team were affiliated with the study organization and familiar with the clinical context. While reflexive discussions and analyst triangulation were used throughout the analytic process to mitigate potential researcher bias, interpretation of the findings may nevertheless have been influenced by researchers’ professional perspectives and contextual knowledge.

As data were collected during the first two years of the COVID-19 pandemic (2021–2022), some contextual factors may have evolved since that time, including changes in public health policy, healthcare system capacity, and community resource availability. However, many of the challenges described by participants, including compassion fatigue, reliance on peer support, and disruptions in community-based services for PEH reflect enduring structural and workforce issues that remain relevant beyond the immediate pandemic context. As such, these findings offer transferable insights for future pandemic preparedness and broader emergency planning.

A further limitation is the difficulty in disentangling stressors associated specifically with caring for PEHs from the broader occupational stress of working in an ED during the COVID-19 pandemic. Some emotional experiences described by participants, such as fear of the unknown, uncertainty, and grief, likely reflect general pandemic-related stressors shared across healthcare settings. However, participants also described challenges more directly linked to caring for PEH, particularly when disruptions in community-based supports increased reliance on the ED for basic needs and social care.

## 5. Conclusions

Strategies to support ED staff as well as institutional and community resources to support the PEHs are warranted in the context of planning for future pandemics. Building psychologically safe workplace environments with supportive colleagues and leaders, is an ongoing process that can support staff resilience to practice optimally during time of added stress such as a pandemic. As highlighted in this study, organizational adaptability and community partnerships focusing on the provision of care for PEH were pivotal to our EDs ability to respond to the needs of this vulnerable population.

This study provides new insight into how caring for PEH during the COVID-19 pandemic shaped the wellbeing of multidisciplinary ED staff, particularly through repeated exposure to housing instability, limited discharge options, and disruptions in community-based supports. These PEH-specific structural challenges contributed to emotional burden, compassion fatigue, and resilience-building experiences across both regulated and unregulated staff roles. Participants identified psychological safety, supportive colleagues, and peer connection as important contributors to coping during periods of heightened workplace stress. Staff also described the importance of organizational adaptability and hospital–community partnerships in supporting continuity of care for PEH when community services were disrupted. These findings suggest that future pandemic preparedness planning should integrate PEH-focused contingency planning alongside staff wellbeing supports, including strengthened hospital–community partnerships to sustain both healthcare delivery and access to essential social supports for structurally vulnerable populations.

## Figures and Tables

**Table 1:** Demographic data for the collective group of respondents who partook in the focus groups. Details on the number of participants per focus group, or which participants were part of each focus group has been omitted to protect the respondents’ privacy.

Gender	Age	Role	Years of Practice	Years at Organization
F	25–29	RN	1–5	1–5
F	50–54	RN	16–20	16–20
F	55–59	RN	<20	<20
M	50–54	Security	<20	16–20
M	35–39	Security	6–10	1–5
F	30–34	Clerical	6–10	6–10
M	35–39	Clerical	6–10	6–10
F	45–50	Clerical	16–20	11–15
F	25–29	Clerical	1–5	1–5
M	40–44	Clinical Assistant	16–20	16–20
F	35–39	Community Support Workers	6–10	1–5
F	30–34	Community Support Workers	1–5	1–5

**Table 6:** Translating study themes into workforce planning and pandemic preparedness actions.

Theme	Key Finding	Actionable Organizational Steps	General or PEH-Specific
Emotional experiences	Staff experienced fear, uncertainty, compassion fatigue and gratitude	Create psychologically safe forums for discussion, regular leadership communication, structured debriefing opportunities	General
Wellness and resilience	Peer support, recognition and self-care supported coping	Implement peer-support initiatives, staff recognition programs, wellness resources and resilience training	General
Hospital-led adaptations	ED space and workflows required rapid modification	Develop contingency plans for rapid space repurposing and surge response	General
Hospital–community adaptations	Loss of community services increased ED reliance among PEH	Establish formal partnerships with shelters, housing providers, primary care, and harm reduction organizations	PEH-specific
Continuity of care for PEH	PEH lost access to essential supports during restrictions	Develop plans for emergency sheltering, temporary housing, transportation and service navigation	PEH-specific
Pandemic preparedness	Need for coordinated planning and communication	Create multidisciplinary pandemic preparedness plans including community partners serving PEH	PEH-specific and General

## Data Availability

The data presented in this study are available upon request from the corresponding author due to privacy.

## Abbreviations

ED	Emergency Department
FGD	Focus Group Discussion
PEH	Persons Experiencing Homelessness
